# Establishing Reference Intervals for Adult Lymphocyte Subsets: Single‐Platform Volume Analysis Reveals Age‐ and Sex‐Specific Trajectories of Immunosenescence

**DOI:** 10.1155/jimr/3532462

**Published:** 2026-09-10

**Authors:** Linjuan Yao, Hui Fang, Shibo He, Jianchao Wang, Xin Jin

**Affiliations:** ^1^ Zhejiang Chinese Medical University, Hangzhou City, Zhejiang Province, China, zcmu.edu.cn; ^2^ Hangzhou Dian Medical Laboratory Center, Hangzhou City, Zhejiang Province, China; ^3^ Department of Clinical Laboratory, Tongde Hospital of Zhejiang Province, Hangzhou City, Zhejiang Province, China, zjtongde.com

**Keywords:** flow cytometry (FCM), immunosenescence, lymphocyte subsets, reference interval

## Abstract

**Background:**

Current clinical reference intervals for lymphocyte subsets lack age‐ and sex‐specific stratification, limiting accurate immune assessment. This study aimed to establish such stratified reference intervals in a large cohort of healthy adults aged 20–60 years.

**Methods:**

A total of 1760 healthy adults were included. The absolute counts and percentage distributions of peripheral blood (PB) lymphocyte subsets were determined via flow cytometry (FCM) using a single‐platform volume method. Differences between age groups and sexes were then compared.

**Results:**

The absolute counts of total T cells and CD8^+^ T cells decreased with advancing age, whereas the absolute counts of CD4^+^ T cells remained relatively stable, although their percentage increased with age. There was a significant difference in immunological age between the 20–40‐year‐old and 41–60‐year‐old groups. Concurrently, the results of this study revealed immunological sexual dimorphism; however, this disparity tended to diminish in the 51–60‐year‐old group because of changes in female sex hormones.

**Conclusion:**

This study established refined reference intervals for adult lymphocyte subsets. The physiological ageing of the immune system constitutes a nonlinear dynamic process, and the establishment of two‐dimensional reference intervals based on both sex and age is recommended.

## 1. Introduction

Peripheral blood (PB) lymphocytes are the principal effectors of the adaptive immune response, and their various subsets have well‐defined, specialised functions in immune surveillance, defence and homeostasis maintenance [1–3]. Therefore, the analysis of PB lymphocyte subsets by flow cytometry (FCM), including the determination of absolute counts and relative proportions, has become an indispensable clinical tool for evaluating an organism’s immune status [4]. More importantly, the applications of this technique have advanced far beyond the traditional diagnosis of immunodeficiency disorders; it is now routinely used in the evaluation of autoimmune diseases, haematological malignancies, immunotherapy for tumours, posttransplant immune monitoring and the assessment of infectious disease progression [5, 6].

Since most reference values do not fully capture the effects of age and sex—two major physiological variables known to profoundly influence the immune system—the accurate interpretation of lymphocyte subsets is hindered. Ageing is invariably accompanied by immunosenescence [7], and sex differences in the intensity of immune responses and susceptibility to autoimmune diseases, which are intimately connected to the immunoregulatory actions of sex hormones, are well documented [8]. Consequently, using a single universal reference value to assess individuals of different ages and sexes can lead to misjudgements regarding normal immune status or conceal early immune imbalances.

The present study has the clear and well‐structured objective of mapping the distribution profiles of major lymphocyte subsets in healthy individuals using FCM with a single‐platform volume method. We aimed to establish refined reference intervals for lymphocyte subsets in healthy adults, stratified by age and sex and ultimately determine their dynamic evolutionary patterns during physiological ageing. These findings will provide a solid basis for the development of an immune age assessment model.

## 2. Materials and Methods

### 2.1. Patient Demographics

Blood samples were collected from 1760 individuals during their physical examinations at the Hangzhou Dian Medical Laboratory Center in 2024. The inclusion criteria were as follows: (1) age ≥20 years and (2) healthy volunteers with normal blood cell counts and no abnormalities in the liver or kidney function. The exclusion criteria included the presence of immune system disorders, haematological disorders, or infectious diseases, as well as the long‐term use of immune‐related drugs. In accordance with the age distribution, the reference population was categorised into various age groups (20–30, 31–40, 41–50 and 51–60 years old).

### 2.2. Specimen Preparation

The sample collection time ranged from 07:00–10:00 AM. The participants fasted and were seated for the collection of antecubital venous blood. Blood samples (2 mL each) were collected in vacuum blood collection tubes containing EDTA‐K2.

### 2.3. FCM Analysis and Reagents

Twenty microlitres of a 6‐colour lymphocyte subset reagent (CD3‐FITC [clone UCHT1]/CD16^+^56‐PE [clones 3G8 and MEM‐188]/CD45‐PerCP‐Cy5.5 [clone HI30]/CD4‐PC7 [clone SK3]/CD19‐APC [clone SJ25C1]/CD8‐APC‐Cy7 [clone SK1] fluorescent monoclonal antibody kit; Beijing Tongsheng Co., Ltd., Beijing, China) and 50 μL of EDTA‐K_2_‐anticoagulated PB were added to a FCM tube. The samples were mixed gently and incubated in the dark at room temperature for 15 min, after which 450 μL of erythrocyte lysis buffer was added. After an additional 10–15 min of incubation at room temperature with mixing, the samples were acquired on a BeamCyte‐1026 M flow cytometer (Mindray Bio‐Medical Electronics Co., Ltd., Shenzhen, China). A minimum of 10,000 CD45+ events were acquired per sample. The gating strategy was as follows: lymphocytes were identified on the basis of CD45 expression and side scatter (SS) properties. Within the lymphocyte gate, T cells were identified as CD3+, B cells as CD19+ and natural killer (NK) cells as CD3−CD16+CD56+. Helper T cells were further identified as CD3+CD4+ and cytotoxic T cells were identified as CD3+CD8+. The complete gating strategy is provided in Figure S1. Absolute counts were obtained using a single‐platform volumetric method, in which the cytometer aspirated a fixed volume and acquired all events within that volume. The absolute count (cells/μL) of each lymphocyte subset was calculated directly by Kaluza software (Beckman Coulter, Inc., Brea, CA, USA) via the CD45 gating strategy without the use of counting beads or external calibrators.

### 2.4. Statistical Analysis

GraphPad Prism 9 (GraphPad Software Inc., San Diego, CA, USA) and SPSS 23.0 software (SPSS Inc., Chicago, IL, USA) were used to analyse the data. The normality of the data distribution was assessed using the Shapiro–Wilk test. Because most immune parameters exhibited a nonnormal (skewed) distribution, reference intervals for absolute lymphocyte subset counts and percentages were established using the nonparametric percentile method, as recommended by CLSI EP28‐A3c guidelines. After excluding outliers using the Tukey method (IQR × 1.5 rule), reference intervals were determined on the basis of the central 95% of the remaining data (i.e., the 2.5th and 97.5th percentiles). Reference intervals were calculated separately for each age group (20–30, 31–40, 41–50 and 51–60 years) and sex.

Comparisons among age groups: For males and females separately, differences in immune parameters across the four age groups (20–30, 31–40, 41–50 and 51–60 years) were assessed using the Kruskal–Wallis *H* test (a nonparametric test for multiple independent samples). When the overall test result was statistically significant, pairwise post hoc comparisons were conducted using the Mann–Whitney *U* test, with a Bonferroni correction applied to control the Type I error rate for multiple comparisons.

Comparisons between sexes: Within each age group, differences in immune parameters between males and females were evaluated using the Mann–Whitney *U* test (a nonparametric test for two independent samples). For parameters that satisfied both the normality and homogeneity of variance assumptions, an independent sample *t*‐test was performed as a sensitivity analysis.

Correlations between variables were calculated using the Spearman rank correlation test. A *p* value <0.05 was considered to indicate statistical significance.

## 3. Results

### 3.1. Normality Testing

A total of 100 immune parameters, stratified by age and sex, were analysed. Among all 100 normality tests performed, only 14 (14%) met the assumption of a normal distribution, whereas the remaining 86% did not follow a normal distribution (*p* < 0.05). Therefore, nonparametric methods were employed for all subsequent analyses.

### 3.2. Age‐Related Differences in Lymphocyte Subsets

**Figure 1:**
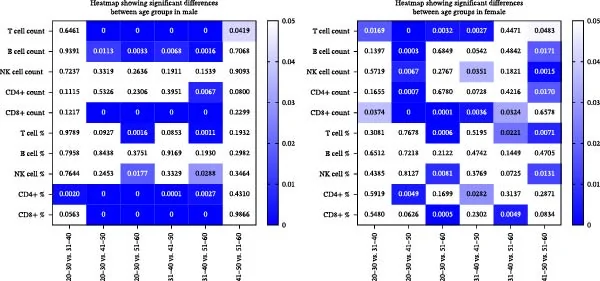
Heatmap showing significant differences in immune parameters across age groups, stratified by sex. The numerical values displayed are *p* values. Differences among the four age groups (20–30, 31–40, 41–50 and 51–60 years) were assessed using the Kruskal–Wallis *H* test. When the overall test was statistically significant, pairwise post hoc comparisons were conducted using the Mann–Whitney *U* test with a Bonferroni correction. Statistical significance was defined as *p* < 0.05. Sample sizes for each group: males: 20–30 years (*n* = 298), 31–40 years (*n* = 330), 41–50 years (*n* = 254), 51–60 years (*n* = 262); females: 20–30 years (*n* = 202), 31–40 years (*n* = 169), 41–50 years (*n* = 127), 51–60 years (*n* = 118).

The correlations between age and lymphocyte subset parameters in the overall population were assessed using Spearman rank correlation analysis (Table 1). The absolute counts and percentages of lymphocyte subsets were compared across the four age groups (20–30, 31–40, 41–50 and 51–60 years) using the Kruskal–Wallis *H* test. Significant age‐related differences were observed for most immune parameters, and the results are summarised in Figure 1.

**Table 1:** Correlation analysis of lymphocyte subsets with age.

Gender	Absolute T cell count	Absolute B cell count	Absolute NK cell count	Absolute CD4+ T cell count	Absolute CD8+ T cell count
Male	*r* = −0.234	*r* = −0.105	*r* = −0.039	*r* = −0.035	*r* = −0.297
	*p* < 0.001	*p* < 0.001	*p* = 0.187	*p* = 0.231	*p* < 0.0001
Female	*r* = −0.172	*r* = −0.078	*r* = −0.018	*r* = −0.061	*r* = −0.197
	*p* < 0.001	*p* = 0.053	*p* = 0.648	*p* = 0.136	*p* < 0.001
Gender	T cell percentage	B cell percentage	NK cell percentage	CD4+ T cell percentage	CD8+ T cell percentage
Male	*r* = −0.110	*r* = 0.031	*r* = 0.080	*r* = 0.206	*r* = −0.218
	*p* < 0.001	*p* = 0.296	*p* = 0.007	*p* < 0.001	*p* < 0.001
Female	*r* = −0.098	*r* = 0.040	*r* = 0.069	*r* = 0.104	*r* = −0.130
	*p* = 0.015	*p* = 0.326	*p* = 0.087	*p* = 0.010	*p* = 0.001

*Note: p* value < 0.05 was considered to indicate statistical significance and was presented in the table. Sample sizes: males: *n* = 1144, females: *n* = 616.

### 3.3. Sex Differences in Lymphocyte Subsets

**Figure 2:**
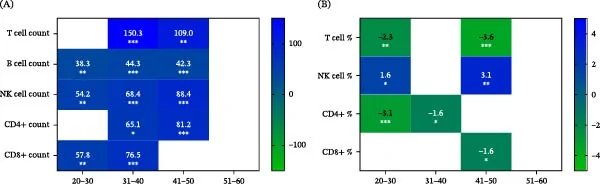
Heatmap of sex‐based differences in immune parameters across age groups. (A) Absolute counts (cells/μL); (B) percentages (%). Positive values (blue) indicate higher median levels in males, whereas negative values (green) indicate higher median levels in females. Statistical significance was determined using the Mann–Whitney *U* test. No sex differences in immune parameters were observed in the 51–60 age group.  ^∗^ *p* < 0.05,  ^∗∗^ *p* < 0.01 and  ^∗∗∗^ *p* < 0.001. Sample sizes for each group: males: 20–30 years (*n* = 298), 31–40 years (*n* = 330), 41–50 years (*n* = 254); females: 20–30 years (*n* = 202), 31–40 years (*n* = 169), 41–50 years (*n* = 127).

Within each age group, differences in immune parameters between males and females were evaluated using the Mann–Whitney *U* test. Comparisons of lymphocyte subset absolute counts and percentages between the sexes are shown in Figure 2.

### 3.4. Interaction Between Age and Sex

**Figure 3:**
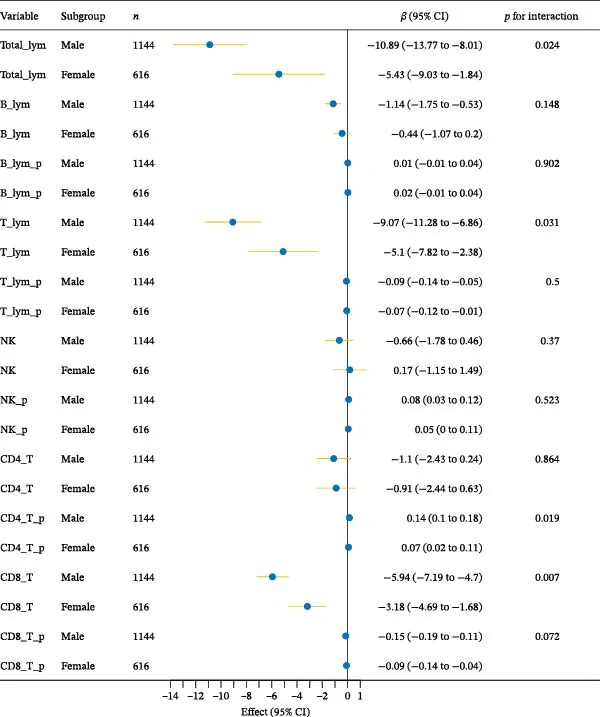
Trends in age‐related changes in different lymphocyte subsets stratified by sex. The results are presented as regression coefficients (*β* values) with 95% confidence intervals (95% CIs); a negative *β* value indicates that the cellular parameter decreases with increasing age, whereas a positive *β* value indicates that it increases with increasing age. *p* values < 0.05 were considered to indicate statistically significant differences. Sample sizes: males: *n* = 1144, females: *n* = 616.

To further explore whether the effects of age on immune parameters differed between sexes, we performed regression analyses, assessed the age‐by‐sex interaction and generated forest plots to visualise the sex‐specific regression coefficients and their 95% confidence intervals (Figure 3). The absolute counts of total lymphocytes, T cells and CD8+ T cells decreased significantly with age in both sexes; however, this decline was significantly more pronounced in males than in females (*p* for interaction = 0.024, 0.031 and 0.007, respectively). Conversely, the percentage of CD4+ T cells increased significantly more with age in males than in females (*p* for interaction = 0.019). No significant age‐by‐sex interactions were detected for B cells or NK cells.

### 3.5. Stratified Reference Intervals for Lymphocyte Subsets

On the basis of the above analyses demonstrating significant age‐ and sex‐related variations, reference intervals for absolute lymphocyte subset counts and percentages were established and stratified by age group and sex using the nonparametric percentile method, as recommended by the CLSI EP28‐A3c guidelines. After excluding outliers using the Tukey method (IQR × 1.5 rule), reference intervals were determined on the basis of the central 95% of the remaining data (2.5th and 97.5th percentiles). The reference intervals for the absolute counts and percentages are presented in Tables 2 and 3, respectively.

**Table 2:** Reference ranges for absolute values of lymphocyte subsets (cells/μL).

Age (years)	Sex	Lymphocyte count	Sex	T cell count	Sex	B cell count	Sex	NK cell count	Sex	CD4^+^ T cell count	Sex	CD8^+^ T cell count
(20–30)	Male (n1 = 298, n2 = 296)	2022 (1063–3157)	Male (n2 = 296)	1415 (681–2314)	Male (n2 = 292)	237 (97–474)	Male (n2 = 282)	332 (125–724)	Male (n2 = 289)	672 (314–1126)	Male (n2 = 291)	604 (268–1047)
	Female (n1 = 202, n2 = 200)	1889 (1098–3035)	Female (n2 = 198)	1351 (748–2144)	Female (n2 = 194)	214 (96–350)	Female (n2 = 196)	289 (105–643)	Female (n2 = 197)	665 (380–1117)	Female (n2 = 200)	550 (290–977)
(31–40)	Male (n1 = 330, n2 = 321)	2003 (1245–3157)	Male (n2 = 319)	1351 (790–2308)	Male (n2 = 322)	234 (98–480)	Male (n2 = 318)	332 (116–759)	Male (n2 = 320)	683 (344–1204)	Male (n2 = 321)	570 (265–1107)
	Female (n1 = 169, n2 = 166)	1813 (1045–2845)	Female (n2 = 163)	1248 (723–1971)	Female (n2 = 163)	198 (96–376)	Female (n2 = 159)	273 (88–574)	Female (n2 = 162)	662 (376–1037)	Female (n2 = 166)	495 (259–997)
(41–50)	Male (n1 = 254, n2 = 252)	1857 (1037–2845)	Male (n2 = 250)	1226 (688–2079)	Male (n2 = 247)	214 (88–421)	Male (n2 = 249)	313 (109–767)	Male (n2 = 248)	684 (288–1197)	Male (n2 = 245)	469 (238–864)
	Female (n1 = 127, n2 = 125)	1611 (1021–2342)	Female (n2 = 126)	1125 (711–1827)	Female (n2 = 122)	185 (91–319)	Female (n2 = 125)	235 (81–571)	Female (n2 = 122)	595 (403–904)	Female (n2 = 125)	448 (207–775)
(51–60)	Male (n1 = 262, n2 = 256)	1768 (939–2643)	Male (n2 = 257)	1132 (600–2025)	Male (n2 = 254)	211 (70–467)	Male (n2 = 250)	327 (100–668)	Male (n2 = 258)	664 (307–1139)	Male (n2 = 251)	439 (176–919)
	Female (n1 = 118, n2 = 116)	1771 (953–2852)	Female (n2 = 116)	1173 (579–2059)	Female (n2 = 116)	204 (76–404)	Female (n2 = 108)	304 (107–674)	Female (n2 = 115)	675 (354–1157)	Female (n2 = 116)	454 (202–1006)
Total	Male (n1 = 1144, n2 = 1125)	1923 (1043–3077)	Male (n2 = 1119)	1292 (668–2196)	Male (n2 = 1118)	223 (88–470)	Male (n2 = 1095)	330 (112–719)	Male (n2 = 1121)	678 (313–1199)	Male (n2 = 1110)	522 (232–1028)
	Female (n1 = 616, n2 = 609)	1762 (1039–2883)	Female (n2 = 601)	1230 (705–2050)	Female (n2 = 597)	201 (93–377)	Female (n2 = 585)	272 (98–610)	Female (n2 = 595)	653 (367–1093)	Female (n2 = 605)	485 (241–969)

*Note:* Excluding outliers using the Tukey method, n1 (pre‐exclusion), n2 (post‐exclusion), data are presented as median (2.5th, 97.5th).

**Table 3:** Reference ranges for percentages of lymphocyte subsets (%).

Age (years)	Sex	T cell percentage	Sex	B cell percentage	Sex	NK cell percentage	Sex	CD4^+^ T cell percentage	Sex	CD8^+^ T cell percentage
(20–30)	Male (n1 = 298, n2 = 298)	69.7 (52.7–81.4)	Male (n2 = 293)	11.8 (5.9–20.3)	Male (n2 = 295)	17.0 (6.0–35.1)	Male (n2 = 297)	33.8 (21.3–45.2)	Male (n2 = 292)	29.6 (18.4–44.3)
	Female (n1 = 202, n2 = 199)	71.7 (58.4–83.1)	Female (n2 = 201)	11.0 (6.1–17.5)	Female (n2 = 199)	15.8 (6.5–30.6)	Female (n2 = 201)	36.6 (25.2–48.7)	Female (n2 = 200)	29.3 (19.9–42.1)
(31–40)	Male (n1 = 330, n2 = 320)	69.7 (53.1–81.2)	Male (n2 = 326)	11.7 (5.9–21.1)	Male (n2 = 325)	17.2 (6.0–36.4)	Male (n2 = 326)	35.2 (21.2–48.0)	Male (n2 = 320)	28.5 (16.7–42.4)
	Female (n1 = 169, n2 = 167)	70.7 (55.1–83.3)	Female (n2 = 163)	10.7 (6.6–18.7)	Female (n2 = 165)	16.5 (4.7–32.4)	Female (n2 = 169)	37.2 (23.0–47.6)	Female (n2 = 168)	28.8 (17.9–43.8)
(41–50)	Male (n1 = 254, n2 = 252)	68.0 (50.2–82.1)	Male (n2 = 251)	11.8 (5.3–21.4)	Male (n2 = 253)	18.0 (5.9–38.7)	Male (n2 = 254)	37.5 (22.8–51.9)	Male (n2 = 253)	25.1 (15.2–43.2)
	Female (n1 = 127, n2 = 125)	70.3 (55.6–85.1)	Female (n2 = 127)	11.4 (5.8–19.1)	Female (n2 = 125)	15.6 (5.9–31.6)	Female (n2 = 125)	38.7 (27.3–51.6)	Female (n2 = 125)	27.7 (17.5–42.3)
(51–60)	Male (n1 = 262, n2 = 259)	67.4 (47.9–82.6)	Male (n2 = 260)	12.1 (4.0–22.6)	Male (n2 = 255)	19.2 (6.0–38.7)	Male (n2 = 260)	37.0 (21.8–53.5)	Male (n2 = 258)	26.1 (13.0–42.4)
	Female (n1 = 118, n2 = 116)	68.7 (52.6–82.0)	Female (n2 = 116)	11.5 (4.4–21.4)	Female (n2 = 117)	18.4 (7.3–35.6)	Female (n2 = 115)	37.4 (24.2–51.7)	Female (n2 = 117)	26.2 (13.1–42.5)
Total	Male (n1 = 1144, n2 = 1134)	68.8 (50.8–82.1)	Male (n2 = 1134)	11.9 (5.4–21.6)	Male (n2 = 1127)	17.7 (5.9–37.3)	Male (n2 = 1134)	35.5 (21.6–49.8)	Male (n2 = 1127)	27.6 (15.2–43.7)
	Female (n1 = 616, n2 = 611)	70.4 (55.8–83.4)	Female (n2 = 602)	11.2 (5.7–18.9)	Female (n2 = 606)	16.3 (6.0–32.5)	Female (n2 = 612)	37.3 (24.3–50.0)	Female (n2 = 611)	28.3 (16.2–42.8)

*Note:* Excluding outliers using the Tukey method, n1 (pre‐exclusion), n2 (post‐exclusion), data are presented as median (2.5th, 97.5th).

## 4. Discussion

The presented data clearly and convincingly demonstrate that the baseline immune system is a dynamic spectrum tightly correlated with an individual’s age and sex rather than a fixed numerical value. Therefore, the percentages and absolute counts of PB lymphocyte subsets vary among different age groups, exhibiting well‐defined age‐related trends alongside important sex differences.

The absolute counts of total T cells and CD8+ T cells decrease with age in both males and females, showing a reliable negative correlation with age. CD8+ T cells, which are the principal effector cells involved in the response to intracellular pathogens and tumour surveillance, undergo a well‐documented decrease in number. This decline is likely attributable to reduced thymic output and clonal expansion driven by prolonged antigen exposure [9]. Repeated antigenic stimulation leads to the dominant expansion of specific clones, while other clones are lost; hence, characteristic oligoclonalisation and numerical depletion of the CD8+ T‐cell pool occur [10]. Consequently, the overall reduction in total T‐cell numbers is primarily the result of the decrease in CD8+ T‐cell counts combined with relatively stable CD4+ T‐cell counts.

The absolute number of CD4+ T cells was not significantly correlated with age in either males or females, but the percentage of these cells was positively correlated with age. Therefore, the stability of CD4+ T‐cell numbers is most likely explained by a more complex and tightly regulated dynamic equilibrium among different functional subpopulations [11]. Because total T‐cell counts decrease with age, whereas CD4+ T‐cell numbers remain relatively stable, the relative proportion of CD4+ T cells among lymphocytes naturally increases. This shift towards a CD4+ relative advantage pattern is the direct cause of the age‐related increase in the CD4+/CD8+ T‐cell ratio, which is now universally recognised as a hallmark of immune senescence and chronic inflammatory states [12, 13].

The different patterns of change observed in B cells and NK cells clearly demonstrate a lack of synchrony between innate and adaptive immune ageing. The age‐related decline in absolute B‐cell counts in males is very plausibly due to an altered bone marrow haematopoietic microenvironment, reduced B‐cell production and impaired germinal centre responses [14]. Notably, the absolute count of NK cells was also low. The data show that this parameter is strongly correlated with age, but its percentage is positively correlated with age in males. This leads to a very clear and elegant conclusion: although the absolute number of NK cells remains relatively constant, their relative proportion increases as the total lymphocyte pool decreases with age (owing to the age‐related decreases in T and B cells). As the rapid‐response force of innate immunity, the relative maintenance of NK cell numbers may therefore partially compensate for the loss of adaptive immune function during ageing [9, 15]; however, their cytotoxic function and cytokine production capacity may still undergo qualitative alterations.

The present study revealed a clear and interesting pattern when age‐stratified reference intervals were constructed: immunological parameters showed very little variation between the 20–30‐year‐old and 31–40‐year‐old groups, and a smooth continuity was observed between the 41–50‐year‐old and 51–60‐year‐old groups. However, when we grouped the 20–30‐year‐old and 31–40‐year‐old age cohorts as the young‐to‐middle‐aged group and compared them pairwise with the 41–50‐year‐old and 51–60‐year‐old cohorts (representing the middle‐to‐older group), statistically significant differences emerged. In the present study, the period from 20 to 40 years of age emerged as a relative plateau phase of immune function, during which no significant age‐related changes were observed in key lymphocyte subsets (Figure 1). These findings are consistent with previous reports suggesting that the peripheral immune system remains relatively stable during early to mid‐adulthood, with more pronounced changes typically occurring after the fourth decade of life [16, 17]. Although thymic atrophy has already begun during this earlier stage, its output function has not yet decreased to a level that would cause a major reduction in the peripheral T‐cell pool; hence, the immune system remains in a relatively stable and efficient state.

The period spanning ~41–50 years of age is widely accepted as the inflection point for immune ageing. Accordingly, many studies point to 40 years of age as the beginning of significant immune changes as thymic output function declines rapidly [18, 19]; consequently, both the proportion and absolute number of naïve T cells decline. Moreover, lifelong chronic antigenic stimulation and chronic inflammation continuously remodel immune homeostasis [20]. This remodelling is not linear or uniform but is relatively concentrated during middle age (41–60 years). This explains why internal variations within the 20–40‐year‐old group were nonsignificant, whereas a distinct ‘immunological age discontinuity’ emerged between this cohort and the 41–60‐year‐old group.

These results have very clear and important implications for clinical practice: conventional 10‐year reference intervals do not adequately capture the biological rhythms that underlie variations in immunological parameters. Hence, we logically recommend that clinical laboratories account for the nonlinear characteristics of immunological ageing when local reference intervals are established and validated. On the basis of our findings, a more scientifically sound and operationally feasible strategy involves dividing adult reference ranges into at least two broad yet biologically distinct age cohorts: young‐to‐middle adulthood (20–40 years), during which immune parameters remain relatively stable, and middle‐to‐old adulthood (41–60 years), when significant age‐related changes become apparent. Because this grouping captures major transitions in immune status without introducing excessive reference range variability that would result from overly fine segmentation, it strikes an ideal balance between practicality and accuracy.

Our research further revealed sex differences in lymphocyte subsets and their evolution with age, painting a dynamic, three‐dimensional interactive portrait of ‘sex–age–immunity’. Specifically, males have higher absolute counts of lymphocytes, T cells, B cells and NK cells. These findings are consistent with previous reports [21] and may be attributed to a combination of factors, including differences in the body surface area, blood volume and the immunomodulatory effects of sex hormones. More importantly, these sex differences demonstrated clear age dependency, with fewer statistically significant differences observed in the 51–60‐year‐old cohort (Figure 2). During reproductive years and early middle age (20–50 years), lymphocyte subsets exhibit complex patterns of quantitative and proportional sex differences, which are likely linked to the active regulatory role of sex hormones. Oestrogen is known to enhance humoral immunity and CD4^+^ T‐cell responses, whereas androgens may exert broader immunosuppressive effects [22, 23]. These hormonal disparities result in women maintaining a higher relative proportion of CD4^+^ T cells than men during this period. However, upon reaching the age range of 51–60 years, significant fluctuations in sex hormone levels occur—particularly in women’s oestrogen levels—and this hormonal withdrawal ultimately erases the previous sex differences in the immune system [24].

Although the general trend of immune ageing can clearly be seen as a progressive decline in absolute counts of lymphocyte subsets, the female cohort showed a striking exception: the absolute counts of T cells, CD4+ T cells and B cells in the 51–60 age group were markedly higher than those in the 41–50 age group, thus forming a clear hump on the immune parameter curve. Importantly, women aged 41–50 years are typically in the perimenopausal phase, during which oestrogen levels begin to fluctuate dramatically but exhibit an overall downward trend. As oestrogen levels decline sharply, the immune system undergoes a transient period of reconfiguration, potentially driving compensatory lymphocyte proliferation in an attempt to establish a new steady state [25, 26]. These findings underscore that establishing clinical reference ranges requires not only the consideration of sex but also its integration with age‐specific factors. Merging reference values for individuals aged 20–50 years with those aged 51–60 years may obscure this physiological transition information. Therefore, when middle‐aged women’s immune function is being assessed, elevated lymphocyte counts in women aged 51–60 years should be interpreted with caution.

Another distinctive feature of this study lies in the methodological approach employed. Rather than utilising the conventional bead‐based method, we adopted a volumetric technique to determine absolute lymphocyte subpopulation counts. The traditional bead‐based method involves the addition of a known quantity of fluorescent microspheres as an internal standard, and the absolute cell count is then indirectly calculated by measuring the ratio of microsphere‐to‐cell events detected via FCM. However, this method introduces potential errors stemming from uneven microsphere‐cell sample processing, microsphere aggregation and instrument variability. In contrast, the volumetric method is a direct, internal‐standard‐free absolute‐counting technique [27]. Its key principle is the precise aspiration of a fixed volume of whole blood during sample preparation for red blood cell lysis and staining; the instrument directly measures and analyses the sample volume to determine the true number of each cell subpopulation per unit volume. The literature clearly supports the conclusion that the volumetric method offers superior accuracy, precision and ease of use. More importantly, it eliminates the systematic biases caused by microsphere batch variations and pipetting errors, yielding results much closer to the gold standard for cell counting [28, 29]. Therefore, the reference ranges established in this study are based on a more accurate volumetric detection technique, making them a far more reliable foundation for absolute count data in both clinical and research contexts. Concurrently, large‐scale age‐ and sex‐stratified reference studies for lymphocyte subpopulations in healthy populations utilising this volumetric method remain lacking.

Notably, our claims regarding differential rates of age‐related changes between males and females were based on formal interaction tests (*p* for interaction), as presented in Figure 3. This approach allowed us to statistically evaluate whether the slope of the age‐related change differed significantly between sexes rather than relying on visual inspection or informal comparisons of correlation coefficients.

Our findings are broadly consistent with previous reports on age‐ and sex‐related differences in lymphocyte subsets in healthy adults [30]. However, our study offers several complementary contributions. First, we employed a volumetric absolute counting method, which eliminates bead‐related biases and provides an alternative reference for laboratories using volumetric platforms. Second, we identified a ‘hump‐shaped’ rebound in female immune parameters during the perimenopausal period (51–60 years), a finding not previously highlighted. Third, we propose a practical two‐tier age stratification (20–40 and 41–60 years) for clinical implementation. These findings complement the literature and provide additional insights into age‐ and sex‐stratified reference intervals.

However, this study has several limitations. First, while it demonstrates age‐related differences, it does not address the longitudinal trajectory of individual immune parameters or their determinants; hence, fingerprint‐based immune function analysis would be more informative. Second, standard FCM phenotyping fails to concurrently reveal cellular functional states and deeper molecular characteristics. We fully agree that a finer classification of lymphocyte subsets (e.g., naïve/memory T cells and activation markers) would substantially increase the biological and clinical relevance of the findings. However, the current study was designed to establish reference intervals based on a routine 6‐colour FCM panel, which did not include markers for naïve/memory subsets or activated antigens. Future studies incorporating naïve/memory T‐cell subsets and activation markers are warranted to further elucidate the immunological mechanisms underlying the age‐ and sex‐related differences observed in this study. Finally, the present study used samples from individuals undergoing routine health examinations. Future research should expand the sample cohort to include populations from different geographic regions and with different occupational exposures. Furthermore, reference ranges should be determined for specific disease states, such as the stable phase of autoimmune diseases and the recovery phase of cancer, to truly maximise the generalisability of the results. For individuals aged 60 years and older, in whom immune ageing progresses more rapidly and interindividual variability is greater, more precise grouping is essential.

In summary, this study systematically delineated the dynamic evolution of the immune system during physiological ageing by establishing age‐ and sex‐specific refined reference intervals for lymphocyte subsets in healthy adults on the basis of a volumetric approach. The investigation highlighted the nonlinear characteristics of immune ageing, revealing a distinct immune age discontinuity between the young‐to‐middle‐aged cohort (20–40 years) and the middle‐to‐old cohort (41–60 years). Furthermore, this study investigated sex differences in immune parameters and their interactions with age. During the reproductive and early middle‐age years (20–50 years), sex differences were pronounced, whereas after 51–60 years of age, immunological disparities between the sexes tended to diminish. Females exhibited a transient hump‐shaped rebound in immunological parameters, emphasising the need to establish two‐dimensional sex–age reference intervals. The volumetric approach employed in this study provides a highly accurate methodological foundation for the detection of absolute cell counts. Despite certain design limitations, the refined reference intervals established here offer crucial standardised data and theoretical underpinnings for the precise clinical assessment of individual immune status and the identification of immune ageing. These findings lay a robust foundation for the future development of personalised immune age assessment models.

## Author Contributions

Linjuan Yao and Xin Jin draughted the manuscript and designed the figures and tables. Linjuan Yao, Hui Fang and Shibo He collected patient information. Linjuan Yao performed the experiments. Jianchao Wang revised the manuscript. Xin Jin and Linjuan Yao conceived the topic. All authors contributed to the article.

## Funding

This work was supported by the Natural Science Foundation of Zhejiang Province (Grant LY23H290003).

## Disclosure

All authors approved the submitted version of the article.

## Ethics Statement

This study was approved by the Ethics Committee of Hangzhou Dian Medical Laboratory Center (Approval Number MR‐33‐25‐073554). Because the study involved only anonymised residual samples from routine health examinations with no additional interventions, the Ethics Committee granted a waiver of written informed consent in accordance with the Declaration of Helsinki.

## Conflicts of Interest

The authors declare no conflicts of interest.

## Supporting Information

Additional supporting information can be found online in the Supporting Information section.

## Supporting information


**Supporting Information** Figure S1: Flow cytometry gating strategy for lymphocyte subset analysis. Lymphocytes were identified by selecting the cell population with low side scatter (SS) and high CD45 expression. Within the lymphocyte gate, T cells were identified as CD3+, and B cells as CD19+. From the CD3− population, NK cells were identified as CD3−CD16+CD56+. Within the CD3+ T cell population, helper T cells were identified as CD3+CD4+ and cytotoxic T cells as CD3+CD8+.

## Data Availability

The data that support the findings of this study are available from the corresponding author upon reasonable request.
