# Supplementary material for: Establishing Reference Intervals for Adult Lymphocyte Subsets: Single‐Platform Volume Analysis Reveals Age‐ and Sex‐Specific Trajectories of Immunosenescence
Source: J Immunol Res. 2026 Sep 10;2026:3532462. doi: 10.1155/jimr/3532462 (PMC13562915; doi:10.1155/jimr/3532462)
Supplement: Supplementary file 1 [file JIMR-2026-3532462-s001.docx]

**Figure S1. Flow cytometry gating strategy for lymphocyte subset analysis.**

Lymphocytes were identified by selecting the cell population with low side scatter (SS) and high CD45 expression. Within the lymphocyte gate, T cells were identified as CD3+, and B cells as CD19+. From the CD3− population, NK cells were identified as CD3−CD16+CD56+. Within the CD3+ T cell population, helper T cells were identified as CD3+CD4+ and cytotoxic T cells as CD3+CD8+.
